# CCN proteins in the musculoskeletal system: current understanding and challenges in physiology and pathology

**DOI:** 10.1007/s12079-021-00631-5

**Published:** 2021-07-06

**Authors:** Veronica Giusti, Katia Scotlandi

**Affiliations:** Laboratory of Experimental Oncology, IRCCS Istituto Ortopedico Rizzoli, 40136 Bologna, Italy

**Keywords:** Bone metastasis, Bone sarcomas, Cellular communication network, Osteoarthritis, Rheumatoid arthritis, Skeletogenesis

## Abstract

The acronym for the CCN family was recently revised to represent “cellular communication network”. These six, small, cysteine-enriched and evolutionarily conserved proteins are secreted matricellular proteins, that convey and modulate intercellular communication by interacting with structural proteins, signalling factors and cell surface receptors. Their role in the development and physiology of musculoskeletal system, constituted by connective tissues where cells are interspersed in the cellular matrix, has been broadly studied. Previous research has highlighted a crucial balance of CCN proteins in mesenchymal stem cell commitment and a pivotal role for CCN1, CCN2 and their *alter ego* CCN3 in chondrogenesis and osteogenesis; CCN4 plays a minor role and the role of CCN5 and CCN6 is still unclear. CCN proteins also participate in osteoclastogenesis and myogenesis. In adult life, CCN proteins serve as mechanosensory proteins in the musculoskeletal system providing a steady response to environmental stimuli and participating in fracture healing. Substantial evidence also supports the involvement of CCN proteins in inflammatory pathologies, such as osteoarthritis and rheumatoid arthritis, as well as in cancers affecting the musculoskeletal system and bone metastasis. These matricellular proteins indeed show involvement in inflammation and cancer, thus representing intriguing therapeutic targets. This review discusses the current understanding of CCN proteins in the musculoskeletal system as well as the controversies and challenges associated with their multiple and complex roles, and it aims to link the dispersed knowledge in an effort to stimulate and guide readers to an area that the writers consider to have significant impact and relevant potentialities.

## Background

CCN proteins were first discovered in the late 1980s and early 1990s as small proteins (30–40 kDa) enriched in cysteine content (10% by mass, 34–38 conserved residues) and characterized by a tetramodular structure. The first reports described them as growth factors due to their ability to control adhesion, proliferation, migration, apoptosis, and extracellular matrix deposition in diverse cell types (for a review see (Perbal 2004)). As research proceeded, CCN members were defined as matricellular proteins, highlighting their secretory nature and their role in conveying/modulating paracrine stimuli by residing in the extracellular matrix (ECM) and interacting with structural proteins, proper signalling factors and cell surface receptors (Rachfal and Brigstock 2005). More recently, the acronym CCN was revised from the historical initials of the first three family members (Cyr61 = CCN1, CTGF = CCN2 and Nov = CCN3) to the novel concept of the cellular communication network, again underscoring the central role of CCN family members in intercellular communication and fine-tuning of the cellular phenotype thanks to their tightly coordinated pattern of expression (Perbal 2018). Cartilage, bone, and skeletal muscles are connective tissues where cells are interspersed in the ECM; in such tissues, which also need to adapt to constant mechanical stimuli, cellular communication mediated by matricellular proteins if of obvious and paramount importance. Indeed, involvement of CCN family members in the physiology of the musculoskeletal system was already identified in the very first reports (Ivkovic et al. 2003; O’Brien and Lau 1992; Wong et al. 1997). Degenerative pathologies of the musculoskeletal system, such as osteoarthritis and rheumatoid arthritis represent a heavy burden on patients’ quality of life and have high social health costs (Martel-Pelletier et al. 2016; Smolen et al. 2018); data on the emerging role of CCN proteins in their pathogenesis may offer therapeutic and regenerative options. Bone sarcomas of the musculoskeletal system, though representing less than 1% of diagnosed tumours per year (Siegel et al. 2021), are highly aggressive cancers that have a tendency for widespread metastasis and often affect children and young adults (Reed et al. 2017). Bone is the preferential site of metastasis of the most common carcinomas in both sexes and bone metastases often lead to skeletal morbidity, such as pathological fracture, pain, spinal cord compression and hypercalcaemia. Skeletal metastasis is associated with reduced patient quality of life, elevated medical costs and ultimately reduced overall survival (Coleman et al. 2020). Matricellular proteins, as CCN family members, have been demonstrated to be involved in every hallmark of cancer (Chong et al. 2012; Yeger and Perbal 2021). The present review aims to provide a comprehensive view of CCN proteins in the development, physiology, pathology, and malignancy of the musculoskeletal system.

## CCN proteins during intrauterine development and differentiation

**Fig. 1:**
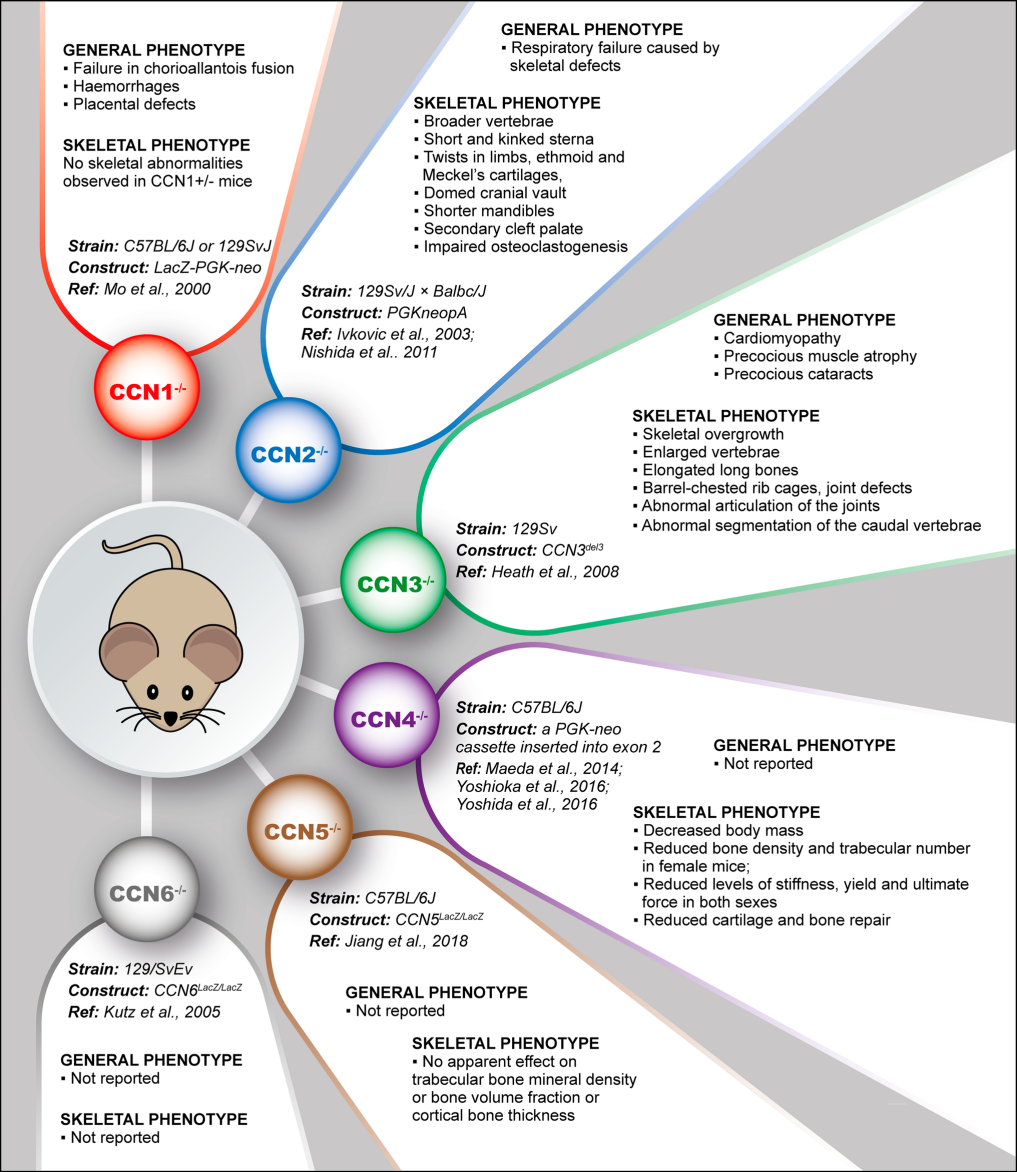
General and skeletal phenotype of mice knockout for different CCN proteins

CCN gene family members are conserved among vertebrates in term of amino acid sequences, protein structures and genetic loci. Bioinformatic analysis revealed an expansion from the sole homologue found in amphioxus, cestode, oyster, and fruit fly to the 4 homologs in lamprey, 5 in ascidian and 9 in zebrafish. Six members belong to this family in mammals, such as mice and humans (Hu et al. 2019) (for a review see Katsube et al. 2009). Given the conservation of the CCN family over evolution, a crucial role of these proteins in vertebrate development can be predicted and has indeed been demonstrated thanks to transgenic mice (Fig. 1) and a description of the expression pattern in the developing musculoskeletal system of vertebrate embryos (Table 1). Only the first two members of the family, CCN1 and CCN2 have been shown to be essential for global embryo development (Ivkovic et al. 2003; Lambi et al. 2012; Mo et al. 2002) and only the expression of CCN2 and CCN3 proved to be essential for correct skeletal development (Heath et al. 2008; Ivkovic et al. 2003; Lambi et al. 2012) (Fig. 1). Therefore, an ancillary role for CCN4, CCN5 and CCN6 in skeletogenesis can be speculated and indeed corresponding knockout mice were viable, fertile, and displayed mild or no skeletal abnormalities (Jiang et al. 2018; Kutz et al. 2005; Maeda et al. 2015). Except for CCN6 (Witte et al. 2009), the expression of all family members has been detected in murine and/or human embryos; CCN2 is expressed solely in active chondrocytes (Friedrichsen et al. 2003; Ivkovic et al. 2003; Kireeva et al. 1997), CCN4 is expressed only in bony elements (French et al. 2004; Witte et al. 2009), and CCN1, CCN3, CCN5 are found in both (Jones et al. 2007; Kanyama et al. 2013; Kireeva et al. 1997; Mo et al. 2002; O’Brien and Lau 1992). Embryonal skeletal muscles expressed low levels of CCN2, CCN3 and CCN5 (Jones et al. 2007; Kocialkowski et al. 2001).

**Table 1:** Expression of CCN family members in murine and human embryonic musculoskeletal system

Protein	Animal model	Expression Pattern in musculoskeletal system				Gestation Period	Technique	Ref
		Cartilage	Bone	Osteoclasts	Skeletal muscle			
CCN1	Mouse	YES peeking E10.5-E14.5;	YES	Not reported	NO	E9.5-E18.5	NB, ISH and IHC	Kireeva et al. 1997; O’Brien and Lau, 1992
	Human	Not reported						
CCN2	Mouse	YES*	NO		YES, weak	E16.5	IHC, ISH	Friedrichsen et al. 2003; Ivkovic et al. 2003; Kireeva et al. 1997
	Human	Not reported	NO	NO	YES, weak	5 months	IHC	Jones et al. 2007
CCN3	Mouse	YES	NO	Not reported	YES	E12-P3	IHC, ISH	Natarajan et al. 2000; Yu et al. 2003
	Human	NO	NO	Not reported	YES	8–10 weeks	NB and ISH	Kocialkowski et al. 2001
CCN4	Mouse	YES*	YES	Not reported	Not reported	E10-E18	ISH and IF and Whole mount ISH	French et al. 2004; Witte et al. 2009
	Human	Not reported						
CCN5	Mouse	YES*	YES	Not reported	YES	E10-E18	ISH and IF and Whole mount ISH	Jones et al. 2007
	Human	Not reported	NO	YES	YES, weak	5 months	IHC	Jones et al. 2007

*High in proliferating, hypertrophic and calcifying zones; not in resting hyaline, mature and semipermanent cartilage

NB: northern blotting; ISH in situ hybridization; IHC immunohistochemistry; IF immunofluorescence

Undifferentiated bone-marrow stem cells express all CCN family members at the mRNA level (Djouad et al. 2007; Schutze et al. 2005). Embryonic and residual adult mesenchymal stem cells (MSCs) are pluripotent precursors defined by the ability to differentiate along adipocytic, chondrocytic and osteocytic lineages. Commitment and cell fate decisions arise from tightly regulated intra- and extracellular signals from both adjacent cells and niche microenvironment. It is therefore not surprising that matricellular CCN proteins under the guise of cellular communication networks are tightly and coordinately modulated during differentiation of MSCs. CCN1 expression decreases during osteogenic differentiation, whereas the expression of the other family members remains constant. During adipogenic commitment CCN1 and CCN5 expression is progressively lost, whereas CCN2 expression slightly decreases (Schutze et al. 2005). Chondrogenic differentiation results from loss of CCN1 expression, decreased CCN2 and CCN6 expression, and upregulation of CCN3 and CCN4 expression (Djouad et al. 2007; Schutze et al. 2005). Many very recent works have also shed light on the pivotal role of CCN2 in tendogenesis. CCN2 promotes clonal expansion, migration and differentiation of the tendon-derived stem cell population synergizing with BMP12 and TGFβ and reversing the detrimental effects of IL1β on collagen deposition (Lee et al. 2015; Liu et al. 2015a, b; Tarafder et al. 2017). CCN2 decreases with age in rat tendon-derived progenitor cells and its exogenous administration rescues these cells from senescence (Lee et al. 2015; Liu et al. 2015a, b; Rui et al. 2019; Tarafder et al. 2017).

Experimental imbalances of CCN protein levels can influence MSC viability. Indeed, exogenous administration of CCN2 decreases the expression of stemness markers (Lee et al. 2010a, b) and CCN4 is required for murine bone marrow-MSC viability as it mediates BMP-3-induced MSC proliferation (Cernea et al. 2016; Schlegelmilch et al. 2014). CCN4 also constitutes a barrier for mouse embryonic cell reprogramming, which requires downregulation of its expression together with that of byglican via upregulation of miR-135b expression (Li et al. 2014). CCN proteins can skew cellular commitment, as well. MSCs derived from CCN1^OCN^ mice, with targeted disruption of CCN1 under the osteocalcin promoter, display reduced osteogenic potential in favour of adipogenic differentiation in vitro (Zhao et al. 2018). Exogenous administration of CCN2 hinders osteogenic and chondrogenic commitment in favour of fibroblastic commitment in human MSCs (Lee et al. 2010a) and skews adipose stem cell toward tendogenic differentiation sustaining proliferation and increasing tendon markers detrimental to expression of osteogenic marker Runx2 (Li et al. 2019). High levels of CCN5 hinder adipogenic differentiation as they dedifferentiate 3T3-L1 adipose cells into pluripotent myofibroblasts expressing α-SMA (Grünberg et al. 2014).

## CCN proteins in the physiology of musculoskeletal system

The vertebrate skeleton is formed through the alternative processes of endochondral and intramembranous ossification (for a review see (Salhotra et al. 2020)). The latter consists of direct differentiation of osteoblasts from MSCs. Endochondral ossification requires the formation of a cartilaginous anlage; during chondrogenesis resting chondroprogenitor cells initially aggregate together to form nodules, proliferate, deposit matrix and terminally differentiate into hypertrophic chondrocytes depositing a calcified matrix. Cartilaginous anlage is later eroded by osteoclasts, multinucleated cells differentiating from macrophages during the process of osteoclastogenesis (for a review see (Xu and Teitelbaum 2013)), allowing invasion of capillaries that convey osteoblast progenitors. Eventually, erosion of cartilaginous anlage and substitution with bone are completed. Endochondral ossification is also a process involved in bone repair. Skeletal muscle cells are also derived from mesenchymal precursors direct differentiation, which requires the proliferation and migration of myoblasts, expressing MyoD and Myf5, subsequent differentiation and expression of specific myogenic markers such as myogenin and MRFs and eventually fusion into myotubes expressing MHC and α-SMA (for a review see (Chal and Pourquié 2017)).

After development, CCN proteins are responsible for maintaining tissue homeostasis in the musculoskeletal system, which undergoes mechanical load and is prone to damage throughout life. These proteins constitute a mechanosensory family, which allows cells to adapt to ever changing conditions modulating the characteristics of the ECM; experimental protocols and cells subjected to mechanical load are extremely varied, indicating that the enhanced expression of CCN proteins upon mechanical stress is likely conserved in the different cytotypes of musculoskeletal system. Notably, in most experimental settings, upregulation of CCN proteins is detected a few hours after mechanical load providing a steady response to changing environment.

### Chondrogenesis

Expression of all CCN family members was induced during experimental chondrogenesis in vitro, with peaks at day 28 or beyond, except for CCN3, which was transiently expressed during only the initial phases of chondrogenesis (Kawaki et al. 2008). Most studies reported below were performed in HCS-2/8 cells, a chondrosarcoma-derived cell line that retains the ability to undergo chondrogenic differentiation and confirmed in primary chondrocytes of both human and animal origin.

**Fig. 2:**
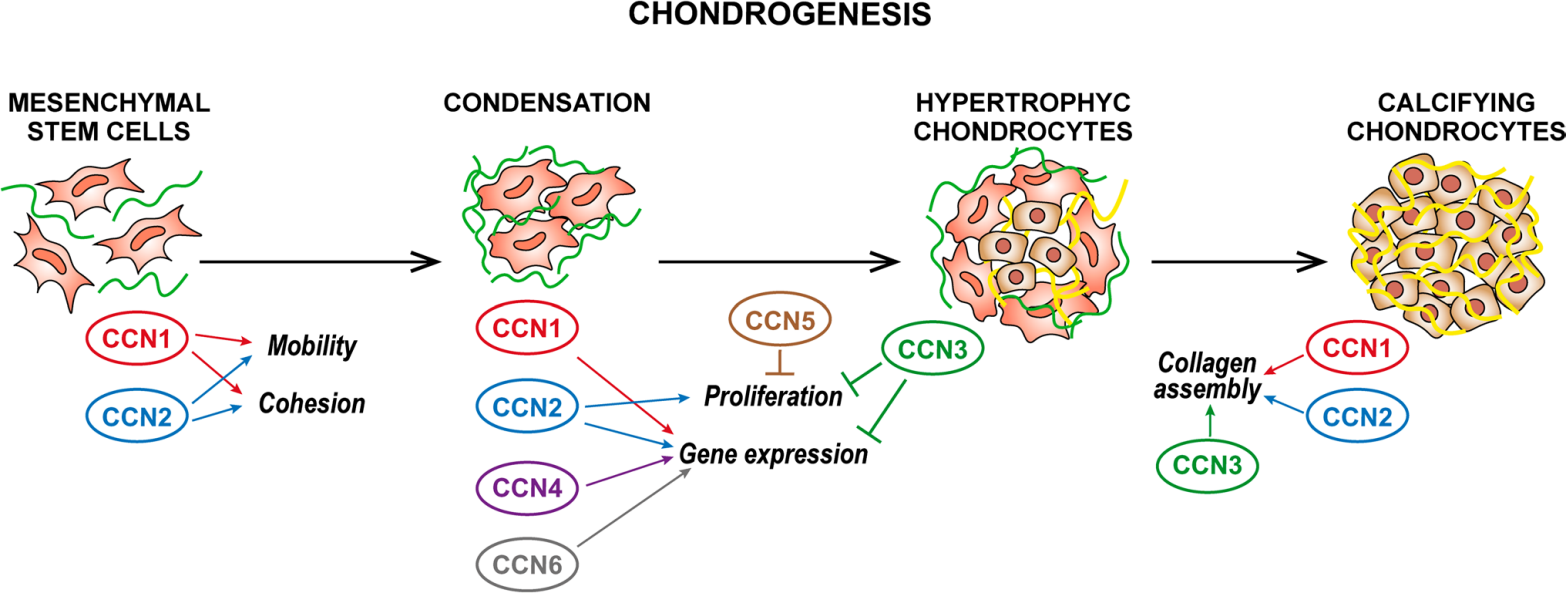
Schematic representation of the role of different CCN proteins in chondrogenesis

CCN1 and CCN2 induce chondrogenesis and promote cell adhesion, cellular aggregation, and node formation, inducing aggrecan and type II collagen with consequent increased proteoglycan synthesis and cartilaginous matrix deposition (Hoshijima et al. 2006; Nishida et al. 2007; Sumiyoshi et al. 2013; Wong et al. 1997) (Fig. 2). A pivotal role of CCN2 in chondrocytes has been proven as this matricellular protein also induces proliferation, DNA synthesis and expression of Sox-9, controls glycolysis and metabolic processes (Maeda-Uematsu et al. 2014; Murase et al. 2016), inhibits ALP activity and protects chondrocytes from apoptosis and autophagy (Fujisawa et al. 2008; Hall-Glenn et al. 2013; Nakanishi et al. 2000; Nakao et al. 2005; Nishida et al. 2002; Xing et al. 2018; Yosimichi et al. 2001). CCN2 is regulated in concert with other CCN family members (Kawaki et al. 2008; Maeda-Uematsu et al. 2014); different growth factors and membrane receptors such as BMP2 and TGFβ (Nakanishi et al. 1997; Parada et al. 2013; Song et al. 2007), M-CSF (Nakao et al. 2005), the actin remodelling pathway (Woods et al. 2009) and FGF1 (El-Seoudi et al. 2017); mir-26a and mir-18a (Etich et al. 2015; Ohgawara et al. 2009); and hypoxic microenvironmental conditions (Nishida et al. 2009). The reader is referred to (Nishida and Kubota 2020) for a more detailed overview of CCN2 role in chondrogenesis. Consistent with its anabolic role in chondrocytes, CCN2 is up regulated in response to mechanical stimuli (Furumatsu et al. 2012, 2013; Nishida et al. 2008). Notably, CCN2 expression is decreased in young chondrocytes exposed to an excessive bout of force (> 15 kPa, 30 cycles/min) and in aged bovine chondrocytes, which consistently show decreased ability to respond to mechanical stimuli (Madej et al. 2016). The anabolic role of CCN2 in chondrocytes can also be exploited for regenerative purposes (Nishida et al. 2004).

CCN4 appears to have a role similar to that of CCN1 and CCN2 in chondrogenesis; its overexpression in MSCs induces chondrogenesis by binding TGFβ3 and exerting synergistic effect during the activation of Smad2/3 pathways leading to enhanced expression of Sox-9, collagen type II and aggrecan (Fig. 2). Consistent with a pro-chondrogenic role, CCN4-/- mouse cartilage displays reduced histological levels of Sox-9, collagen type II and aggrecan as well as reduced repair after full thickness damage (Yoshioka et al. 2016). According to another report, CCN4 synergizes with Wnt3a in inhibiting TGFβ-induced Smad2/3 pathways in P2 cells and human G6 chondrocytes and skewing cellular phenotype towards osteogenesis (van den Bosch et al. 2014). The alternative role of CCN4 may depend on the upstream activating pathway as well as expression of splicing variants detected in HCS-2/8 cells (Yanagita et al. 2007).

CCN3 counteracts the proliferative potential of CCN2 as it keeps terminally differentiated chondrocytes in a quiescent state and its exogenous administration, overexpression or in vivo supplementation inhibits chondrocyte proliferation and proteoglycan deposition and induces type II collagen, type X collagen, tenascin, lubricin and TGFβ2 expression, whereas its silencing yields the opposite results (Janune et al. 2011b, 2017; Kawaki et al. 2008; Lafont et al. 2005b) (Fig. 2). CCN3 plays a different role in murine costal and articular chondrocytes, undergoing intramembranous ossification, where it increases proteoglycan synthesis (Janune et al. 2011a).

Far less is known about CCN5 and CCN6 during chondrogenesis. CCN5 overexpression in primary chondrocytes reduces cell viability and PPARγ activation as observed in developmental dysplasia of the hips (DDH) (Ji et al. 2020) (Fig. 2). CCN6 expression increases in adult tissues suggesting a role in cartilage maintenance (Sen et al. 2004): it induces type II collagen and Sox-9 expression and weakly binds and antagonizes IGF-1 induced hypertrophy a collagen type X and Runx2 expression and ALP activity (Cui et al. 2007; Repudi et al. 2013; Sen et al. 2004) (Fig. 2). Loss of CCN6 function, due to at least 70 known mutations, causes progressive pseudo rheumatoid dysplasia (PPD), a rare autosomal disease characterized by symmetrical polyarticular involvement without systemic inflammation, knobbly interphalangeal joints of the hands, and gait abnormalities (for a review see (Torreggiani et al. 2019)). The role of CCN6 in disease pathogenesis is largely unknown, also due to the lack of skeletal phenotype in CCN6 knockout mice (Kutz et al. 2005).

### Osteogenesis and fracture healing

**Fig. 3:**
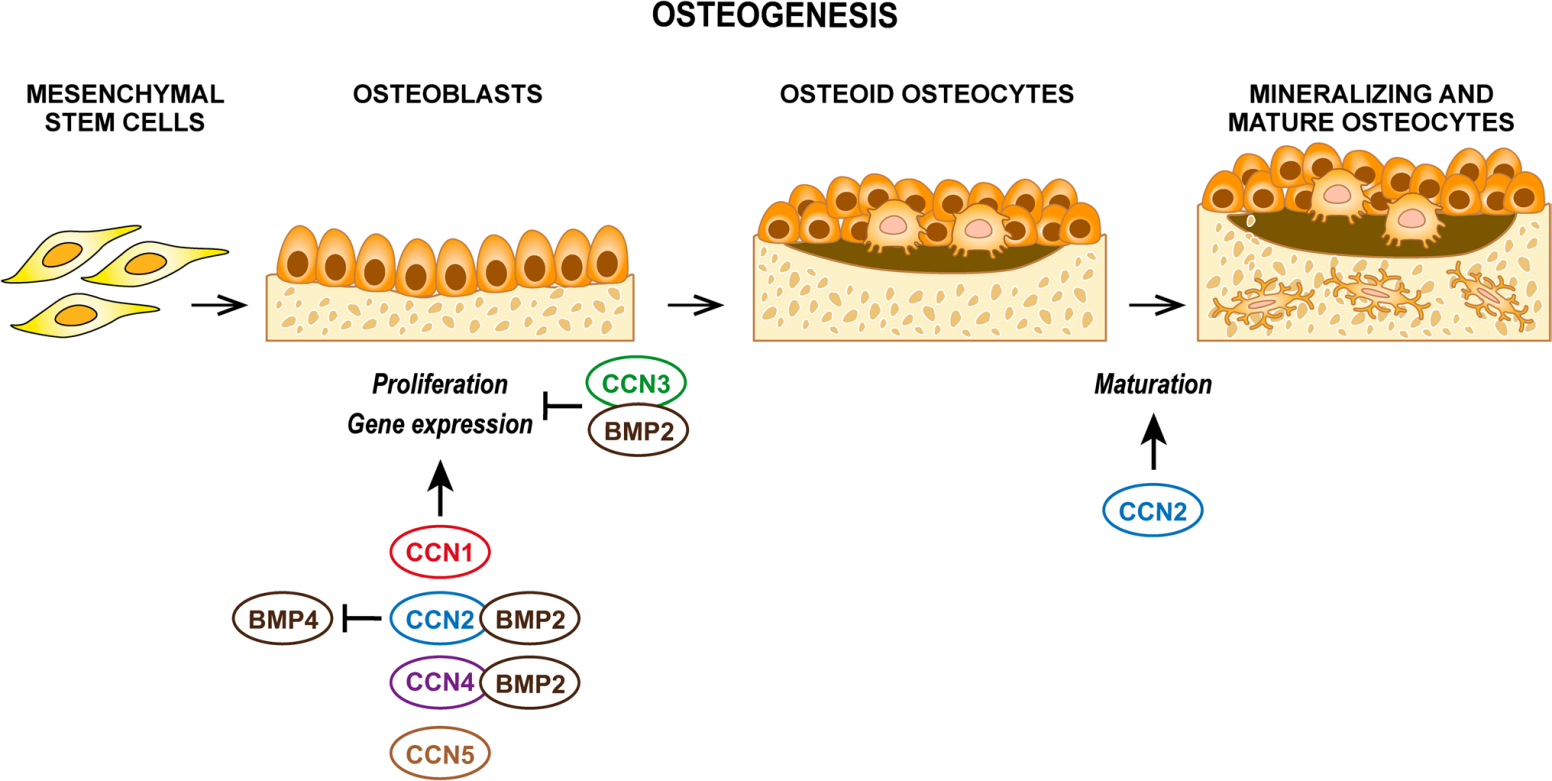
Schematic representation of the role of different CCN proteins in osteogenesis

All CCN family members were detected in murine bone tissues, with CCN6 being the least expressed protein: CCN1, CCN2, CCN3 and CCN6 were observed only on bone surfaces, whereas CCN4 and CCN5 were detected in bone matrix as well (Kawaki et al. 2011). CCN1, CCN2, CCN4 expression peaks at days 14–28 during experimental osteogenesis in vitro, whereas CCN3 expression steadily decreases. CCN5 expression slightly increases during osteogenesis in vitro; exogenous CCN5 has no effect on osteoblast proliferation, but induces osteoblast maturation by upregulating Osterix, ALP and Bsp via Smad 1/5/8, β-catenin and p38 mediated pathways (Kawaki et al. 2011) (Fig. 3). The expression of CCN6 is very low during osteogenesis and its exogenous administration or silencing has no effect on osteogenesis in vitro (Kawaki et al. 2011).

CCN1 and CCN2 promote proliferation and differentiation inducing the expression of type I collagen, Runx2 and Bsp via Smad 1/5/8, β-catenin and p38 mediated pathways (Kawaki et al. 2011) (Fig. 3). Osteoblast-specific CCN1 deletion in CCN1^OCN^ mice leads to decreased mineral density, bone volume-to-total volume ratios, trabecular bone surface and area, and decreased peak and fail force (Zhao et al. 2018). As observed for chondrogenesis, CCN2 also plays a more profound role in osteogenesis, with more potent effects on osteoblast proliferation, adhesion, and differentiation and enhancement of ALP expression and activity, osteocalcin, osterix and osteopontin resulting in enhanced formation of mineralized nodules in vitro (Kawaki et al. 2011; Nishida et al. 2000; Safadi et al. 2003; Smerdel-Ramoya et al. 2008; Wang et al. 2009). CCN2 is again regulated in concert with osteogenic factors: CCN2 mediates the osteogenic potential of Notch, preptin and Wnt signalling and its expression is induced by TGFβ, BMP2, IGF-1, cortisol, and taurine in osteoblasts (Maeda et al. 2009; Nakanishi et al. 2000; Pereira et al. 2000; Yuan et al. 2007), antagonizes BMP4 inhibitory action on osteoblasts (Smerdel-Ramoya et al. 2008) and is in turn antagonized by PPARγ and TNFα (Ni et al. 2020; Yu et al. 2014). Upregulation of CCN2 is observed in skeletal pathologies such as ossification of the posterior longitudinal ligament (OPLL) (Yamamoto et al. 2002) and spondylocarpotarsal syntosis (SCT) (Zieba et al. 2016). Consistently, both CCN1 and CCN2 are overexpressed in the first phases of bone healing and in osteoblasts under mechanical stress (Hadjiargyrou et al. 2000; Hu et al. 2012; Kadota et al. 2004; Kanyama et al. 2013; Kulkarni et al. 2012; Lienau et al. 2006). CCN4 plays a more limited role in osteogenesis; it has no osteogenic potential per se, but directly binds BMP2 and promotes its binding to integrin receptors in MSCs with a synergistic effect on BMP-promoted osteogenesis (Inkson et al. 2009; Kawaki et al. 2011) (Fig. 3). CCN4 expression is induced in temporary calluses in structures linking new to old bone upon injury (French et al. 2004) and in human preosteoblastic CIMC-4 cell line upon uniform biaxal 2% strain (Case et al. 2008). Consistent with a pro-osteogenic role, CCN4 knock in mice display denser bone in females, and CCN4-/- mice fail to repair fractures (French et al. 2004).

As observed for chondrogenesis, CCN3 has opposite effects to CCN2 and inhibits osteogenesis by directly binding to BMP2 and inhibiting its downstream signalling (Kawaki et al. 2011; Minamizato et al. 2007; Rydziel et al. 2007) (Fig. 3). Consistently, mice overexpressing CCN3 in osteoblasts, under the osteocalcin promoter, show decreased mineral density and bone formation rates (Rydziel et al. 2007). CCN3 expression increases upon drilled hole femoral injury in osteogenic cells (Matsushita et al. 2013).

### Osteoclastogenesis

**Fig. 4:**
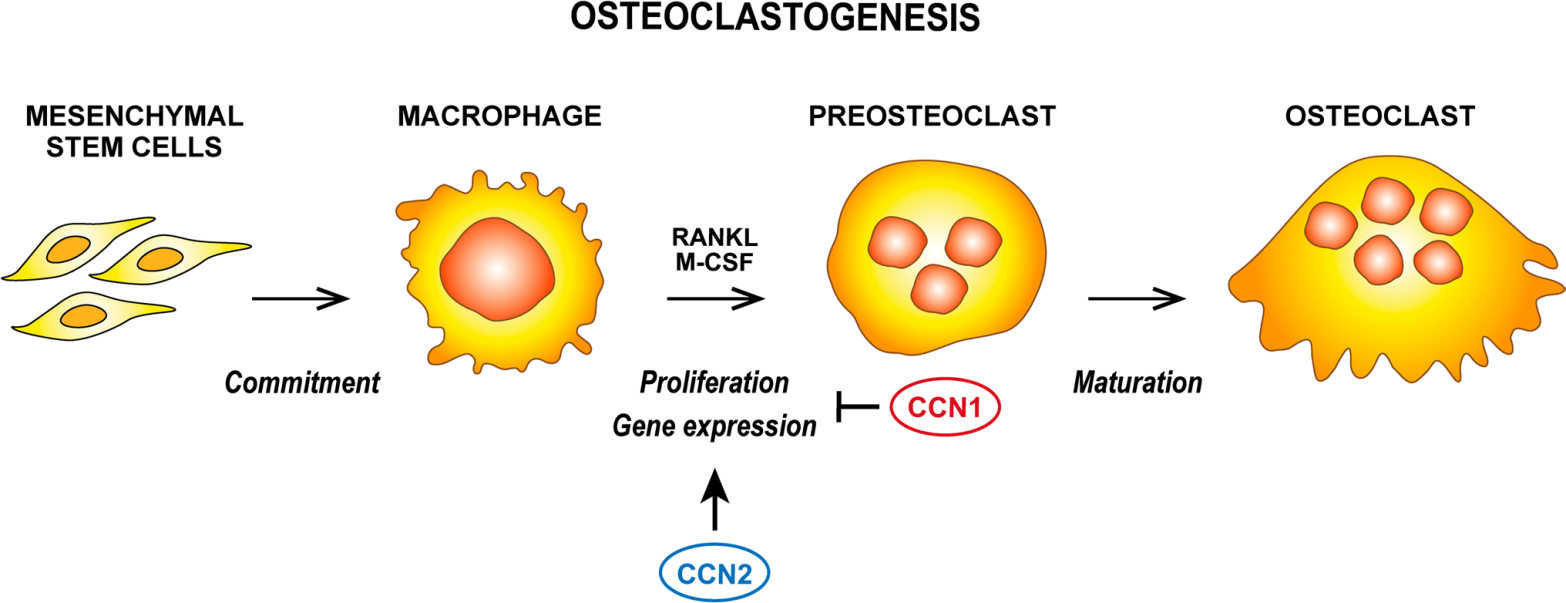
Schematic representation of the role of different CCN proteins in osteoclastogenesis

Knowledge concerning CCN family members in osteoclastogenesis is limited to CCN1 and CCN2, that play contrasting roles (Fig. 4).

CCN1 inhibits osteoclastogenesis in murine, rabbit and human macrophages stimulated with M-CSF and RANKL; it decreases TRAP expression and activity as well as the expression of integrins, calcitonin receptors, cathepsin K and MMP-9. The effects of CCN1 are not mediated by induction of apoptosis, inhibited cell fusion, or alterations in the p38, ERK and NF-kB pathways. Moreover, the number and activity of mature osteoclasts are not repressed by CCN1 (Crockett et al. 2007).

CCN2 has no osteoclastogenic potential per se, but it favours RANKL-induced osteoclastogenesis in RAW 264.7 macrophages with pleiotropic activities. It synergizes with RANKL in inducing NF-kB translocation into nuclei, activating p38 and inducing NFATC1 and c-Fos; it inhibits Bcl6, an osteoclastogenesis repressor, and favours cell fusion via DC-STAMP (Aoyama et al. 2015; Nishida et al. 2011, 2019) and it counteracts anti-osteoclastogenic effect of osteoprotegerin (Aoyama et al. 2015). The pivotal role of CCN2 in osteoclastogenesis is further corroborated by downregulation of miR-26a, which would inhibit CCN2 itself (Kim et al. 2015).

Consistently, CCN1^OCN^ and CCN2 overexpressing mice show an increased number and activity of osteoclasts, together with upregulation of RANKL and inhibition of osteoprotegerin (Zhao et al. 2018). In contrast, fewer osteoclasts are identifiable in the bones of CCN2-/- mice and stimulation with RANKL and M-CSF in foetal liver cells fails to induce osteoclastogenesis (Nishida et al. 2011).

### Myogenesis

All CCN family members, except CCN5 and CCN6, were found to be expressed during myogenesis; for a detailed description of the role of CCN proteins in the physiology of the skeletal muscle the reader is also referred to (Rebolledo et al. 2021).

**Fig. 5:**
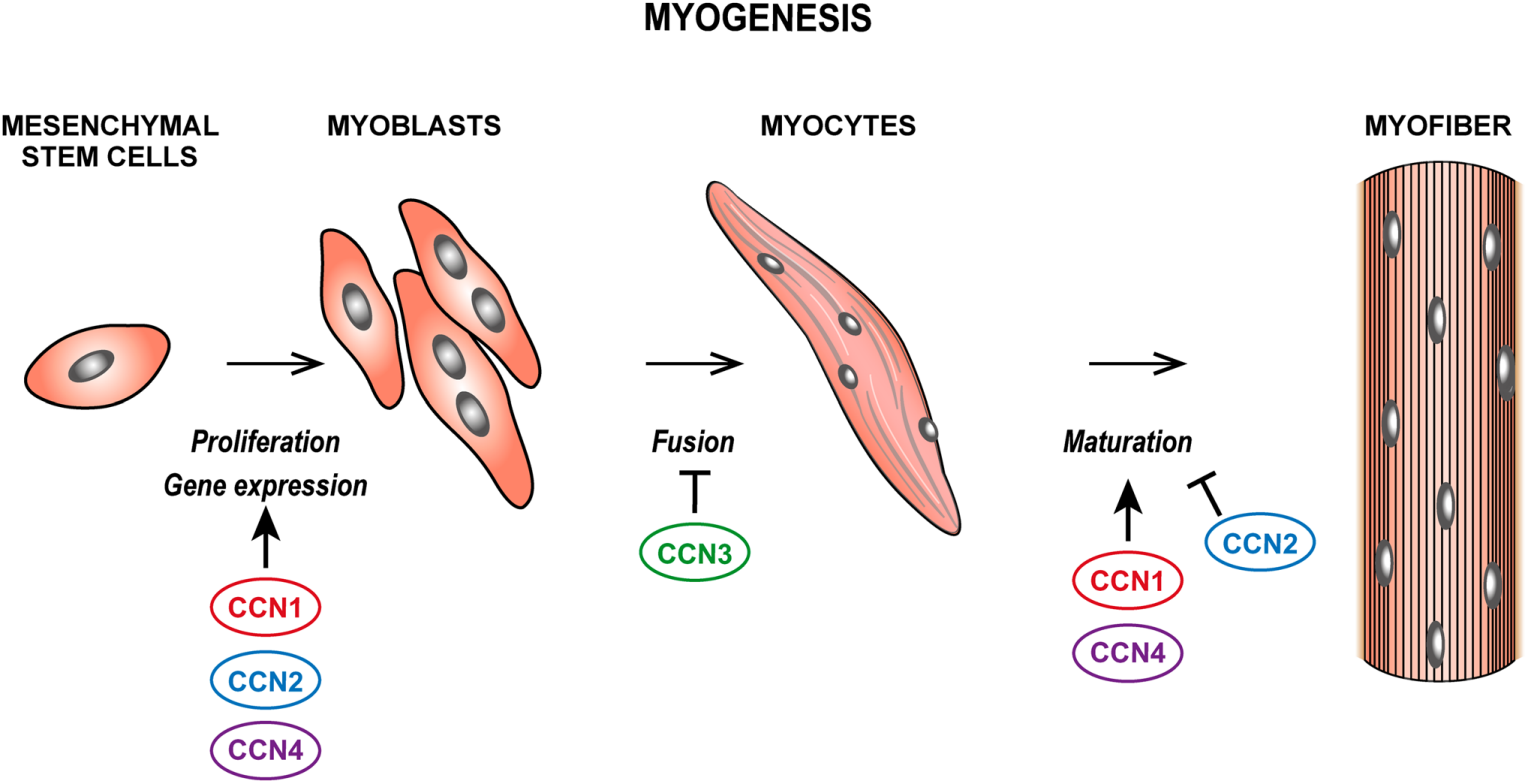
Schematic representation of the role of different CCN proteins in myogenesis

CCN1 is expressed at all stages of myogenesis in vitro and in vivo under the control of NFATC5, which in turn fosters myoblast migration and differentiation (O’Connor et al. 2007) (Fig. 5). Similarly, upon induction by TGFβ1 and LPA, CCN2 stimulates myoblasts proliferation, upregulates fibronectin, integrins α4, α5, α6 and β1 and MyoD but represses markers of terminal differentiation of myocytes (Nishida et al. 2015) (Fig. 5). CCN1 and CCN2 expression is also up regulated in human skeletal muscles of individuals following a jumping exercise (Kivelä et al. 2007) and downregulated following muscle denervation (Magnusson et al. 2005), thus indicating a possible role in physiologically working muscles.

CCN4 also displays myogenic activity; its expression increases as myoblasts differentiate and its inhibition decreases the expression of myosin heavy chain, myogenin, MRF and MEF-2 (Wang et al. 2008) (Fig. 5). Consistently, CCN4 knockout mice display lower expression of myogenic markers and reduced myofiber repair; moreover, aged osteoblasts expressing less CCN4 fail to sustain muscle stem cell proliferation and differentiation (van den Bosch et al. 2017).

CCN3 is reported to mediate myoblast adhesion in a calcium and integrin-dependent manner and to augment myoblast sensitivity to IGF-1, FGF2 and PDGF (Lafont et al. 2005a) but its overexpression in C2/4 cells hinders myotube formation (Sakamoto et al. 2002) (Fig. 5).

## CCN proteins in degenerative and inflammatory diseases of the musculoskeletal system

Degenerative and inflammatory diseases of the musculoskeletal system severely limit patients' quality of life and have high medical costs. CCN family member deregulation has been identified in both osteoarthritis and rheumatoid arthritis and offers therapeutic insights (for a review see (MacDonald et al. 2021)). Osteoarthritis (OA) is an age-related degenerative disease of the articular cartilage, that progressively becomes eroded and substituted by fibrotic tissue (for a review see (Martel-Pelletier et al. 2016)). Rheumatoid arthritis (RA) is an inflammatory disease of joints. The synovium is infiltrated by inflammatory cells, which cause oedema, hyperplasia of the synovial lining, and degradation of the ECM (for a review see (Smolen et al. 2018)). CCN2 has also been ascribed a central role in intervertebral disc disease (IVD) which is the main cause of low back pain in adults (Matta et al. 2018; Wang et al. 2018): CCN2 downregulation associated with aging (Hyiama et al. 2018) and CCN2 notochord-specific knockout leads to precocious and more pronounced degeneration (Bedore et al. 2013).

Moreover, CCN2 is found in the cytoplasm of necrotic and regenerating fibres and in active fibroblasts in the endomysial space of dystrophic human muscles and in mdx mice affected by Duchenne muscular dystrophy (DMD) (Morales et al. 2018; Song et al. 2017; Sun et al. 2008). CCN2 overexpression has proven detrimental for muscle fibres as it transiently augments the expression of fibronectin, collagen type III and decorin and diminishes isometric specific and tetanic forces as well as the response to BaCl_2_ damage (Morales et al. 2011). Consistently, genetic CCN2 inactivation in mdx mice increases the running ability of mice, reducing necrosis, myogenin and myosin heavy chain (MHC) expression, and collagen stiffness via downregulation of Lox (Petrosino et al. 2019); also, the effects of ACE inhibitors on dystrophy are mediated by a decrease in CCN2 expression (Morales et al. 2013). For further insights into the role of CCN proteins in pathology and regeneration of the skeletal muscle, the reader is also referred to (Rebolledo et al. 2021).

### Osteoarthritis

CCN family members have been found to be differentially expressed in the superficial cartilage layer and matrix of OA patients, with maximal expression levels for CCN2, moderate expression levels for CCN1 and CCN4, low expression level for CCN3 and CCN5 and no expression for CCN6 (Komatsu et al. 2015).

CCN1 and CCN2 concentrations in sera and/or synovial fluid are positively correlated with disease severity (Chijiiwa et al. 2015; Honsawek et al. 2012; Komatsu et al. 2015). Monoiodoacetate intra-articular (MIA)- injected mice, an experimental model of OA, also show upregulation of CCN2 expression (Nishida et al. 2004). The role of CCN1 in OA pathogenesis is mainly related to the promotion of inflammatory response as it is induced upon TGFβ, TNFα and IL1β stimulation in osteoarthritic chondrocytes and HCS-2/8 cells (Chijiiwa et al. 2015; Moritani et al. 2005). CCN2 participates in OA pathogenesis upregulating IL1β and VEGF expression through inhibition of miR-210 (Liu et al. 2015b) and the establishment an autocrine loop with M-CSF fostering osteoclastogenesis (Nakao et al. 2005). The role of CCN2 in OA is corroborated by elevated expression of lncRNA PVTI targeting miR-26b, which would in turn inhibit CCN2 (Ding et al. 2020). OA is ameliorated by CCN1 inhibition through siRNA in a model of gouty arthritis induced by injection of monosodium urate crystals or through berberine in a collagen-induced OA (CIOA) murine model via downregulation of IL6, TNFα and IL1β expression via NF-kB pathway (Zhou et al. 2020). On the contrary, OA is ameliorated by additional CCN2 expression, which promotes cartilage repair; local administration via a CCN2-loaded hydrogel fosters reparation of articular cartilage through upregulation of tenascin-C, aggrecan and type X collagen expression (Nishida et al. 2004), and the beneficial effects of hyaluronan are mediated by induction of CCN2 expression in human synovial cells (Lee et al. 2010b). Consistently, the appearance of osteoarthritic-like degeneration is delayed up to 21 weeks in CCN2 knock-in mice: increased cellularity and decreased proteoglycan loss are derived from reduced collagen type X and MMP13 and collagen type I expression in articular chondrocytes (Itoh et al. 2013). Similarly, CCN4 is up regulated in synovial tissues fluid of OA patients and the damaged cartilage of OA experimental models (van den Bosch et al. 2019). CCN4 polymorphisms are associated with spinal OA in Japanese women (Urano et al. 2007). Detrimental effects of CCN4 overexpression include both direct effects on cartilage such as decreased matrix component synthesis and deposition, increased matrix degradation, due to augmented expression and activity of aggrecanase and metalloproteinase, and proinflammatory effects such as upregulation of IL6 and monocyte adhesion through upregulation of VCAM-1 expression (Hou et al. 2013; van den Bosch et al. 2015, 2019).

CCN3 expression shows a positive correlation with age in both mice and humans (Kuwahara et al. 2020). The role of CCN3 in cartilage degeneration is cumbersome; both mice overexpressing CCN3 and mice with targeted disruption of CCN3 show precocious degenerative changes (Kuwahara et al. 2020; Roddy and Boulter 2015). Findings in osteoarthritis confirm this dualism. Indeed, CCN3 expression leads to p53 accumulation in nuclei and p21 promoter activation and hence correlates with the expression of senescence markers, together with the expression of proinflammatory cytokines IL-6 and IL-8 and decreased expression of aggrecan (Kuwahara et al. 2020). On the other hand, CCN3 protects articular cartilage from damage as it antagonizes IL1β-induced upregulation of aggrecanase, MMPs, and iNOS expression and induction of autophagy in chondrocytes by blocking HMGB1 and TLR4. Nevertheless, IL1β has itself been shown to be capable of diminishing CCN3 expression in both SW1353 cells and rat primary chondrocytes (Huang et al. 2019).

### Rheumatoid arthritis

All CCN family members were found to be up regulated in RA sera and tissues (Komatsu et al. 2015), but only the role of CCN1 and CCN2 in its pathogenesis has been fully explored. CCN3 expression correlates with disease activity and IL6 production (Wei et al. 2020) and CCN4 single nucleotide polymorphisms have been associated with the risk of developing RA and response to therapy (Kuo et al. 2019).

Both CCN1 and CCN2 are up regulated in synovial tissues, fibroblast-like synoviocytes and sera of RA patients (Chen et al. 2017a, b; Ding et al. 2016; Jie et al. 2015; Nozawa et al. 2009; Zhai et al. 2017; Sun et al. 2020; Zhu et al. 2015). A CCN1 serum concentration higher of 99.66 pg/ml (Fan et al. 2019) and a CCN2 higher than 73.35 pg/ml (Yang et al. 2017) correlate with disease. CCN1 and CCN2 have similar roles in RA pathogenesis, as both favour fibroblast-like synoviocite proliferation and induce osteoclastogenesis as well as angiogenesis (Chen et al. 2017a, 2017b; Ding et al. 2016; Ganesan and Rasool 2017; Nozawa et al. 2009; Zhang et al. 2009). Moreover, CCN1 induces the expression of the proinflammatory cytokine IL-6 (Choi et al. 2020; Lin et al. 2012), IL8 and CCL20 which promote neutrophil and monocyte migration to the synovium (Chen et al. 2017a; Zhu et al. 2015), and MMPs, which foster cell migration (Kwon et al. 2017).

Given their pleiotropic role in RA pathogenesis, a multitude of efforts have been made to inhibit CCN1 and CCN2 with a great diversity of approaches. Targeted gene editing (lentishCCN1, AdshCCN1) and administration of anti-CCN1 monoclonal antibodies, or drugs (rosiglitazone) in collagen-induced (CIA) murine models of RA invariably led to an amelioration of RA symptoms with decreased inflammatory scores and monocyte infiltration, decreased production of proinflammatory cytokines such as IL-6 and IL1β and increased production of antinflammatory IL10, decreased MMP activity and osteoclastogenesis with reduced RORc, TNFα, IL17 and sRANKL levels, and ultimately reduced osteoclast populations (Chen et al. 2017a, b; Kwon et al. 2017; Lin et al. 2012; Zhai et al. 2017; Zhu et al. 2015). Blocking pathways upstream of CCN1 induction, such as IL17 via cyanidin or ferulic acid, or TNFα via SIRT1 or simvastatin, also led to amelioration of RA symptoms (Ganesan and Rasool 2017; Kok et al. 2013; Samarpita et al. 2020). In addition to CCN1, CCN2 targeting by monoclonal antibodies in CIA mice reduced the incidence and symptoms of RA, Th17 population and osteoclastogenesis, T cell proliferation and MMP3 activity (Nozawa et al. 2013; Nozawa 2014).

## CCN proteins in sarcomas of the musculoskeletal system

CCN proteins actively participate in carcinogenesis, as their role has been clarified in every hallmark of cancer except inflammation (for a review see (Yeger and Perbal 2021)). Nevertheless, the role of at least CCN1 and CCN2 in rheumatoid arthritis underscores their association with the regulation of proinflammatory cytokines in the microenvironment. Thus, CCN proteins are involved in cancer in a pleiotropic manner and particularly serve sarcomas of the musculoskeletal system.

### Osteosarcoma

Osteosarcoma is the most frequent malignancy of bone and commonly affects children and young adults. CCN1, CCN2, CCN3 and CCN4 are all poorly expressed in healthy adult bony tissues, but they are up regulated in primary samples as well as osteosarcoma cell lines (Chen et al. 2013a; Fromigue et al. 2011; Habel et al. 2019; Huang et al. 2016; Liu et al. 2017b; Perbal et al. 2008; Tsai et al. 2014a, 2014b, 2014c; Wu et al. 2013; Zhang et al. 2013). The pivotal roles of CCN1 and CCN2 in osteosarcoma are corroborated by the decreased expression of miRNAs inhibiting their expression, such as miR-33a, miR-100 and miR-365, and miR-584, respectively (Huang et al. 2014, 2018; Li et al. 2020; Xu et al. 2018); on the other hand, miR-25-3p which indirectly fosters CCN1 and CCN2 expression is up regulated in osteosarcoma (Rao et al. 2020). CCN1 and CCN2 do not promote osteosarcoma cell proliferation per se but protect cells from apoptosis and decrease chemotherapeutic efficacy (Chen et al. 2013a; Fromigue et al. 2011; Habel et al. 2015, 2019; Huang et al. 2016; Tsai et al. 2014a, 2014b, 2014c; Zhang et al. 2013). A plethora of in vitro and preclinal models have ascertained the roles of CCN1 and CCN2 in promoting migration and invasiveness. CCN1 induces MMP-2, MMP-9, MMP-14 and TIMP-3, mediates prometastatic effects via the IGF-1/IGF1Rβ pathway, and is elevated in MG63 M7 and M10 sublines selected in vivo for a migratory phenotype; in addition, its inhibition decreases lung-metastatic potential of osteosarcoma cell lines in vivo (Chen et al. 2013a; Habel et al. 2015, 2019; Hou et al. 2014). CCN2 promotes osteosarcoma cell migration by downregulating miR-519d and upregulating MMP-2 and MMP-3 (Hou et al. 2018; Tsai et al. 2014c). CCN4 also promotes migration, invasiveness and metastasis of osteosarcoma cells upregulating MMP-2 and MMP-9 expression (Tsai et al. 2017; Wu et al. 2013). Moreover, CCN1 and CCN4 have proangiogenic activity promoting expression of VEGFA (Habel et al. 2015; Kim et al. 2018; Liu et al. 2017b). Last, CCN1 promotes epithelial-to-mesenchymal transition (EMT) skewing cells towards a spindle-like morphology and a more staminal and migratory phenotype (Habel et al. 2019; Hou et al. 2014). CCN3 deserves separate mention as its role in osteosarcoma is controversial. On the one hand, consistent with the antiproliferative role in osteocytes, its upregulation in 143B cells with poor CCN3 expression decreases cell vitality, proliferation and clonogenicity, and its silencing in MG63 cells yields the opposite effect (Huang et al. 2011). On the other hand, CCN3 has been associated with increased migration and upregulation of COX-2 expression (Huang et al. 2011; Yao et al. 2015) and CCN3 expression correlates with poor prognosis, high expression of MRP1-4 and resistance to cisplatin, doxorubicin, and methotrexate (Perbal et al. 2008).

### Chondrosarcoma

Chondrosarcoma is the second most common malignancy of bone and is characterized by poor response to therapy and metastatic phenotype. CCN1, CCN2, CCN3 and CCN4 are expressed in enchondromas and low-grade chondrosarcomas, whereas their expression diminishes as disease progresses to higher grades and less differentiated lesions (Hou et al. 2011; Shakunaga et al. 2000; Tan et al. 2009a, 2009b; Yu et al. 2003). CCN6 expression is also up regulated in chondrosarcoma with respect to healthy tissues and its expression correlates with a metastatic phenotype (Fong et al. 2012). Consistent with their role in osteosarcoma, CCN proteins expression foster migration in chondrosarcoma by augmenting expression and enzymatic activity of MMP-13 (Tan et al. 2009a, 2009b; Tzeng et al. 2011), MMP-2 (Hou et al. 2011) or ICAM-1 (Fong et al. 2012).

### Ewing sarcoma

Ewing sarcoma is an aggressive malignancy of bone that often develops in children, molecularly characterized by translocation most commonly involving the EWS and FLI1 genes. CCN1, CCN2 and CCN3 are expressed in all Ewing cell lines, though heterogeneously (Perbal et al. 2009; Strammiello et al. 2003). As observed in osteosarcoma, the role of CCN3 in Ewing sarcoma is controversial. Its exogenous expression in TC-71 cells blocks proliferation, clonogenicity and the cell cycle, increases apoptosis and decreases tumorigenic potential in vivo (Benini et al. 2005). Nevertheless, as observed in osteo- and chondrosarcoma, CCN3 promotes TC-71 cell migration and invasion, decreasing adhesion on collagen type I and IV via integrin α2β1 (Benini et al. 2005). Consistently, CCN3 expression correlates with higher metastatic potential in clinical samples (Perbal et al. 2009).

### Rhabdomyosarcoma

Rhabdomyosarcoma is a malignancy of skeletal muscles and represents the most common soft tissue cancer in infancy. CCN2 is expressed in spontaneous rhabdomyosarcomas of the genitourinary tract of Balbp53neu transgenic mice (Croci et al. 2007) and human rhabdomyosarcoma cell lines of both alveolar and embryonal subtypes (Croci et al. 2004). CCN2 inhibition hinders growth and induces apoptosis, though diminishing myogenic differentiation, in accordance with the role of CCN2 in myogenesis (Croci et al. 2004).

## Bone metastasis of other cancers

**Fig. 6:**
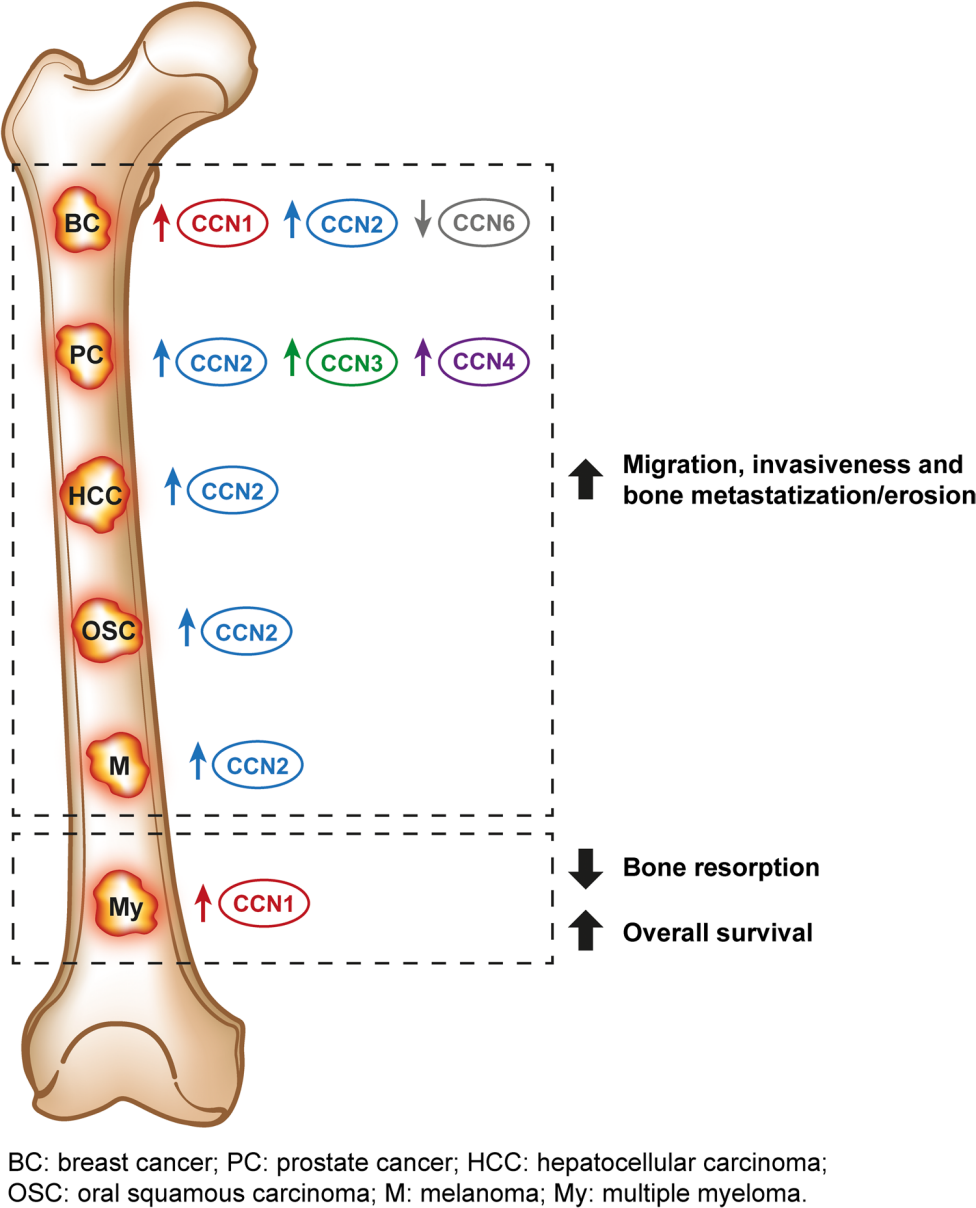
Schematic representation of the role of different CCN proteins in bone metastasis of other cancers

Bone is a preferential site of metastasis for breast cancer (70%) and prostate cancer (85%) and myeloma (95%) (Coleman et al. 2020). Metastatic dissemination of tumour cells requires adaptation to the unique milieu of specialized bone cells, mineralized bone matrix: CCN proteins, as cellular communication networks, mediate this heterologous interaction, thus generally favouring bone invasion and metastatic cells proliferation with the exceptions CCN6 in breast cancer and CCN1 in myeloma (Fig. 6). In this field, the most studied family member is CCN2 which also figures in Kang’s bone metastasis signature for genes cooperating in bone metastasis development irrespective of the primary tumour site (Casimiro et al. 2012). In addition to frequent bone-metastatic cancer, discussed below, CCN2 is highly expressed in bone invasive oral squamous carcinoma (Shimo et al. 2008), melanoma (Braig et al. 2011) and bone metastasis of hepatocellular carcinoma in both murine models and clinical samples (Hou et al. 2015; Xiang et al. 2011, 2012), where high CCN2 expression positively correlates with rate of bone metastasis (Xiang et al. 2011; Zhang et al. 2019).

### Breast cancer

CCN1 and CCN2 are up regulated in breast cancer and their expression correlates with staging and local (CCN1) or bone (CCN2) invasiveness (Kim et al. 2020; O’Kelly et al. 2008). Forced CCN1 expression in MCF7 low-invasive breast cancer cell line increases its ability to adhere to collagen and fibronectin, invasiveness on fibronectin substrate and migratory ability (O’Kelly et al. 2008). Comparison of different breast cancer cell lines or parental cells with those experimentally enriched for metastatic potential, invariably highlighted overexpression of CCN2 (Kim et al. 2020; Ohgawara et al. 2011). CCN2 cooperates in angiogenesis and osteoclastogenesis induced by metastatic breast cancer cells, inducing angiopoietin 2 and RANKL (Kim et al. 2020). Consistently, its silencing diminishes invasive potential, MMP-9 and MMP-13 expression and activity, and Runx2 expression in vitro (Ren et al. 2014) and an anti-CCN2 monoclonal antibody reduces metastatic potential of MDA-MB-231 upon intracardiac injection with remaining metastases displaying a less angiogenetic and osteoclastic phenotype (Shimo et al. 2006). In contrast with the prometastatic role of CCN1 and CCN2, CCN6 acts as an oncosuppressor in breast cancer, preserving epithelial differentiation. CCN6 silencing in human mammary epithelial cells (HME) leads to loss of acinar polarity, clear cell–cell borders, decreased E-cadherin and integrin α6 expression and failure to form a lumen, thus favouring a less epithelial and more invasive phenotype. Conversely, forced CCN6 expression in MDA-MD-231 and SUM149 cells reverts the migratory phenotype and restores the ability to form an acinar lumen. Consistently, CCN6 expression is downregulated in human breast cancer (Pal et al. 2012); for a review of CCN6 role in breast cancer see (Tran and Kleer 2018).

### Prostate cancer

CCN2, as observed in breast cancer, CCN3 and CCN4 levels are up regulated in prostate cancer and bone-metastatic cells or corresponding bone metastasis (Dankner et al. 2019; Kim et al. 2020; Ono et al. 2013). Consistently, exogenous administration of CCN2 prior to in vivo injection increases number and size of bone metastases, whereas its inhibition reduces bone metastasis and related osteosclerosis (Kim et al. 2020; Zhang et al. 2018). CCN3 in conditioned medium from prostate cancer cells favours osteoclastogenesis via both RANKL-dependent and RANKL-independent pathways in vitro and forced overexpression of CCN3 in PCa and LnCAP C4-2 cells enhances osteolytic potential and TRAP-staining in metastatic lesions (Chen et al. 2013b). CCN4 promotes adhesion of prostate cancer cells to bone via upregulation of VCAM1 and integrin α4β1 expression on osteoblasts and migration of prostate cancer cells themselves (Chang et al. 2018; Tai et al. 2014). Inhibition of CCN4 by monoclonal antibodies reduces metastatic burden and in particular metastases to bone versus other localizations reducing migration and invasiveness (Ono et al. 2013).

### Myeloma

CCN1 is up regulated in multiple myeloma tissues as well as in patients’ sera (Johnson et al. 2014; Liu et al. 2017a). Contrary to what observed for other CCN proteins and cancers, its expression positively correlates with better prognosis; consistent with its inhibitory role in osteoclastogenesis, exogenous administration of CCN1 to multiple myeloma cells in coculture with MSCs slows proliferation and bone resorption, favouring osteoblasts to the detriment of osteoclasts (Johnson et al. 2014; Liu et al. 2017a).

## Concluding remarks

CCN1 and CCN2 play pivotal stimulating roles in development, physiology, pathology, and malignancy of the musculoskeletal system. CCN4 has a minor role in all aspects whereas CCN3 counteracts the actions of CCN2. The roles of CCN5 and CCN6 in the musculoskeletal system remain poorly studied and unclear. Efforts should be made to perform a more coordinated study of the effects of these family members as a team rather than single entities in the physiology of both osteoclasts and skeletal muscle cells as well as degenerative diseases and cancer. Regenerative approaches could also benefit from considering the integrated role of CCN family members in fine-tuning the function of diverse tissues and cell types in the musculoskeletal system. Lessons learned from the proinflammatory role of CCN1 and CCN2 in rheumatoid arthritis should prompt studies on the ability of CCN family members to affect the tumour microenvironment and immune evasion. Differential expression between osteosarcomas/bone metastasis and surrounding normal bone or between low- and high-grade chondrosarcomas offers a rationale for developing targeted therapeutics, at least for young adult patients. Last, given the involvement of CCN2 in bone metastasis regardless of the of primary tumour site, its level in biological fluid could provide a precocious marker of skeleton related events.

## Abbreviations

CCN1-6: Cellular communication network 1–6
ECM: Extra-cellular matrix
mRNA: Messenger RiboNucleic Acid
BMP-2, 3, 4, 12: Bone morphogenetic protein 2, 3, 4, 12
TGFß1-3: Transforming growth factor ß 1–3
IL1β, 6, 8, 10, 17: Interleukin 1β, 6, 8, 10, 17
Runx 2: Runt-related transcription factor 2
α-SMA: Smooth muscle α -actin
MyoD: Myogenic differentiation antigen
Myf5: Myogenic Factor 5
MRF: Myogenic regulatory factors
MHC: Myosin heavy chain
DNA: DesossicriboNucleic Acid
Sox-9: SRY-Box Transcription Factor 9
ALP: Alkaline phosphatase
M-CSF: Macrophage colony-stimulating factor
FGF 1, 2: Fibroblast Growth Factor 1‚ 2
Smad 1/2/3/5/8: Small mothers against decapentaplegic 1/2/3/5/8
Wnt3a: Wingless/Integrated 3a
PPARγ: Peroxisome Proliferator Activated Receptor γ
IGF: Insulin-Like Growth Factor 1
Bsp: Bone Sialoprotein
p38: Mitogen-activated protein kinases
TNF α: Transforming growth factor α
(s)RANKL (soluble): Receptor Activator of Nuclear Factor Kappa B Ligand
NF-kB: Nuclear Factor Kappa Beta
NFATC1, 5: Nuclear factor of activated T cells 1, 5
Bcl6: B-cell lymphoma 6 protein
TRAP: Tartrate-Resistant Acid Phosphatase
MMP 1, 2, 3, 9, 3, 14: Matrix metalloproteinase 1, 2, 3, 9, 13, 14
ERK: Extracellular Signal-Regulated Kinase
DC-STAMP: Dendritic Cell-specific Transmembrane Protein Allergy
MEF-2: Myocyte enhancer factor 2
PDGF: Platelet-derived growth factor
Lox: Lectin-like oxidized low-density lipoprotein (LDL) receptor
ACE: Angiotensin Converting Enzyme
VEGF: Vascular endothelial growth factor
lncRNA: Long non coding RiboNucleic Acid
PVT1: Human Plasmacytoma Variant Translocation 1
siRNA: Silencing RiboNucleic Acid
VCAM-1: Vascular cell adhesion molecule 1
P21: Cyclin-dependent kinase inhibitor 21
iNOS: Inducible Nitric Oxide Synthase
HMGB1: High mobility group box 1
TLR4: Toll-like receptor 4
CCL20: Chemokine (C–C motif) ligand 20
RORc: RAR related orphan receptor C
SIRT1: Sirtuin (silent mating type information regulation 2 homolog) 1
TIMP-3: Metalloproteinase inhibitor 3
IGF1Rβ: Insulin like growth factor 1 receptor β
MRP1-4: Multidrug resistance-associated protein 1–4
ICAM-1: Intercellular Adhesion Molecule 1
